# Statistics of Cholera

**Published:** 1856-10

**Authors:** 


*Statistics of Cholera.*—We introduce the following statistics of the treatment of cholera with some degree of confidence in their general results. Treatment, however, of all departments of statistical medicine, is the most uncertain, and affords the least assistance in the presence of disease. In prescribing for a patient, it is unnecessary to say, that we are not bound to give him the treatment which affords the best *average* of success. It may happen that the mode generally least successful is specially adapted to the case in hand:

